# Clinical symptoms in mild cognitive impairment with Lewy bodies: Frequency, time of onset, and discriminant ability

**DOI:** 10.1111/ene.15783

**Published:** 2023-03-21

**Authors:** Paul C. Donaghy, Calum Hamilton, Rory Durcan, Sarah Lawley, Sally Barker, Joanna Ciafone, Nicola Barnett, Kirsty Olsen, Michael Firbank, Gemma Roberts, Jim Lloyd, Louise M. Allan, Ranjan Saha, Ian G. McKeith, John T. O'Brien, John‐Paul Taylor, Alan J. Thomas

**Affiliations:** ^1^ Translational and Clinical Research Institute Newcastle University Newcastle Upon Tyne UK; ^2^ Nuclear Medicine Department Newcastle Upon Tyne Hospitals NHS Foundation Trust Newcastle Upon Tyne UK; ^3^ Centre for Research in Ageing and Cognitive Health University of Exeter Exeter UK; ^4^ Department of Psychiatry, School of Clinical Medicine University of Cambridge Cambridge UK

**Keywords:** Alzheimer disease, dementia, dementia with Lewy bodies, diagnosis, mild cognitive impairment

## Abstract

**Background and purpose:**

Mild cognitive impairment with Lewy bodies (MCI‐LB) is associated with a range of cognitive, motor, neuropsychiatric, sleep, autonomic, and visual symptoms. We investigated the cumulative frequency of symptoms in a longitudinal cohort of MCI‐LB compared with MCI due to Alzheimer disease (MCI‐AD) and analysed the ability of a previously described 10‐point symptom scale to differentiate MCI‐LB and MCI‐AD, in an independent cohort.

**Methods:**

Participants with probable MCI‐LB (*n* = 70), MCI‐AD (*n* = 51), and controls (*n* = 34) had a detailed clinical assessment and annual follow‐up (mean duration = 1.7 years). The presence of a range of symptoms was ascertained using a modified version of the Lewy Body Disease Association Comprehensive LBD Symptom Checklist at baseline assessment and then annually.

**Results:**

MCI‐LB participants experienced a greater mean number of symptoms (24.2, SD = 7.6) compared with MCI‐AD (11.3, SD = 7.4) and controls (4.2, SD = 3.1; *p* < 0.001 for all comparisons). A range of cognitive, parkinsonian, neuropsychiatric, sleep, and autonomic symptoms were significantly more common in MCI‐LB than MCI‐AD, although when present, the time of onset was similar between the two groups. A previously defined 10‐point symptom scale demonstrated very good discrimination between MCI‐LB and MCI‐AD (area under the receiver operating characteristic curve = 0.91, 95% confidence interval = 0.84–0.98), replicating our previous finding in a new cohort.

**Conclusions:**

MCI‐LB is associated with the frequent presence of a particular profile of symptoms compared to MCI‐AD. Clinicians should look for evidence of these symptoms in MCI and be aware of the potential for treatment. The presence of these symptoms may help to discriminate MCI‐LB from MCI‐AD.

## Graphical abstract

A range of cognitive, parkinsonian, neuropsychiatric, sleep, and autonomic symptoms were significantly more common in mild cognitive impairment (MCI) with Lewy bodies (LB) compared to mild cognitive impairment due to Alzheimer disease (AD). A previously defined 10‐point symptom scale demonstrated very good discrimination between MCI‐LB and MCI‐AD (area under the receiver operating characteristic curve = 0.91, 95% confidence interval = 0.84–0.98), replicating our previous finding in a new cohort.

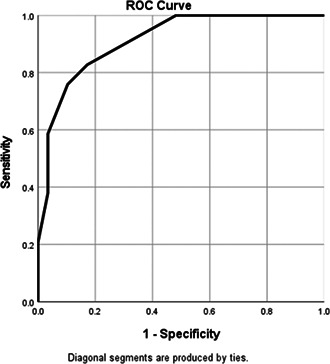

## INTRODUCTION

Dementia with Lewy bodies (DLB) affects both the central and peripheral nervous systems and is associated with a range of features including cognitive, motor, neuropsychiatric, sleep, autonomic, and visual symptoms [1, 2]. Research criteria for the diagnosis of prodromal DLB were published in 2020, including operationalized criteria for the diagnosis of mild cognitive impairment with Lewy bodies (MCI‐LB) [3]. Similar diagnostic entities are also defined in the current versions of the Diagnostic and Statistical Manual of Mental Disorders (5th edition) and the International Classification of Diseases (11th revision) [4, 5]. There has been an associated increase in interest in MCI‐LB and other forms of prodromal DLB in recent years. It is vital that we understand the clinical presentation of MCI‐LB, to understand the symptoms that patients are likely to experience and the utility of clinical symptoms to differentiate between MCI‐LB and other forms of MCI, particularly MCI due to Alzheimer disease (MCI‐AD). Simple scales to help identify MCI‐LB in the clinic would be of use to researchers and clinicians.

Data reporting the prevalence of a range of symptoms in MCI‐LB have started to emerge [6, 7, 8, 9, 10], including our report in 2017, which investigated the cross‐sectional prevalence of a range of symptoms based on the original Lewy Body Disease Association Comprehensive LBD Symptom Checklist [11]. We identified 10 symptoms that were common in DLB (>50%) and relatively rare in AD (<20%) and demonstrated that this combination of symptoms discriminated between MCI‐LB and MCI‐AD. Since the initial report, we have followed these patients longitudinally and have recruited a further, independent sample of participants with MCI‐LB and with MCI‐AD and controls. The objectives of this study were to:

1. Investigate the cumulative frequency of symptoms from baseline assessment to the point of dementia diagnosis in both cohorts combined, and compare rates between MCI‐LB and MCI‐AD.
2. Analyse the discriminant ability of the 10‐point symptom scale to differentiate between MCI‐LB and MCI‐AD in an independent cohort.

## METHODS

### Participants

Participants ≥60 years old with MCI were recruited from memory clinics, older people's medicine clinics, and neurology clinics in North East England and Cumbria from February 2013 to September 2019 as part of two studies, LewyPro and SUPErB. Participants were identified by clinical staff or research staff embedded in the clinical team, or from research case registers. LewyPro recruited participants from February 2013 until February 2016. SUPErB recruited participants from March 2016 until September 2019. The "Longitudinal Cumulative Prevalence" section of the results reports data from both studies. The "Cross‐Sectional Replication of 10‐Symptom Discrimination" section of the results reports data only from participants who were not part of the LewyPro study, to replicate the discriminant ability of the 10‐point symptom scale that was developed in the LewyPro cohort.

Potential participants were approached if they experienced symptoms that might be related to prodromal DLB, such as autonomic symptoms, visual disturbances, olfactory impairment, and mood changes as well as any indication of the presence of core and supportive features of DLB. Participants were excluded if they had a diagnosis of dementia, an Mini‐Mental State Examination score < 20, a Clinical Dementia Rating score > 0.5, parkinsonism that developed >1 year prior to cognitive impairment, or evidence of clinical stroke or a serious neurological or medical condition that would affect their performance in study assessments. Participants with a current episode of major depression or a history of bipolar disorder or schizophrenia were also excluded. In the SUPErB study, participants with symptomatic heart failure (New York Heart Association Class II or greater) were excluded to avoid false‐positive cardiac metaiodobenzylguanidine (MIBG) results [12].

All participants gave their written informed consent to take part in the study. The study received ethical approval from the National Research Ethics Service Committee North East–Newcastle & North Tyneside 2 (research ethics committee identification numbers 15/NE/0420, 12/NE/0290).

### Assessment

Participants had a comprehensive clinical assessment as detailed previously [13, 14].

During their clinical assessment, participants (with a relative, friend, or carer present where possible) were asked whether they experienced a range of symptoms, adapted from a previous version of the Lewy Body Disease Association Comprehensive LBD Symptoms Checklist (Table S1). An updated version of this is freely available online at https://lbda.org/wp‐content/uploads/2020/09/comprehensive_lbd_symptom_checklist_2019.pdf. The questionnaire was administered at baseline and follow‐up assessments. The mean duration of follow‐up was 1.7 years (SD = 1.4).

The interviewer asked whether each symptom was present. When participants reported a symptom, they were asked how long it had been present.

Ten symptoms were identified in our previous paper as being common in DLB (>50%) and uncommon in AD (<20%) and were found to accurately discriminate between MCI‐LB and MCI‐AD in our previous report [11]. The number of these 10 symptoms that were present was recorded for each participant.

### Diagnosis

An expert consensus clinical panel (A.J.T., P.C.D., J.‐P.T.) reviewed all the clinical assessment data to confirm subjects met NIA‐AA all‐cause MCI criteria [15] without considering aetiology. Where the first two raters did not agree, the third made a final decision. The consensus panel also rated the presence or absence of each of the four core symptoms of DLB (cognitive fluctuations, complex visual hallucinations, motor parkinsonism, and clinical rapid eye movement [REM] sleep behaviour disorder [RBD]). These symptoms were evaluated with specific scales during the clinical assessment (the Dementia Cognitive Fluctuations Scale [16], the Clinician Assessment of Fluctuation Scale [17], the North East Visual Hallucinations Interview [18], the Unified Parkinson's Disease Rating Scale motor scale [19], and the Mayo Sleep Questionnaire [20]). Parkinsonism was defined as the presence of bradykinesia, a parkinsonian rest tremor, or rigidity, as set out in the MCI‐LB criteria [3]. The symptom ratings were combined with imaging biomarker results where available (^123^I‐FP‐CIT (Ioflupane) single photon emission computed tomography and cardiac ^123^I‐MIBG (meta‐iodobenzylguanidine)) to classify participants as probable MCI‐LB (two core clinical features or one core clinical feature and at least one abnormal MCI‐LB biomarker), possible MCI‐LB (one core clinical feature or one abnormal MCI‐LB biomarker), or MCI‐AD (none of the four core features and no abnormal MCI‐LB biomarkers and evidence of decline consistent with AD with no evidence for another aetiology) [3, 15].

The "1‐year rule" was applied so that no subjects had had evidence of parkinsonism for >1 year before the onset of their cognitive decline. Cerebrospinal fluid (CSF) and imaging biomarkers were not used in the diagnosis of MCI‐AD; therefore, the MCI‐AD cases fulfilled the NIA‐AA "Core Clinical Criteria" for MCI‐AD. Assignment to these diagnostic categories was based on information from both baseline and follow‐up clinical evaluations where available. Participants were included if their final diagnosis was probable MCI‐LB, probable DLB [2], MCI‐AD, or AD [21]. Participants with a final diagnosis of probable MCI‐LB or probable DLB will be referred to as "MCI‐LB" in this article. Participants with a final diagnosis of MCI‐AD or AD will be referred to as "MCI‐AD."

### Statistics

Statistical comparisons were performed using SPSS and SAS software. MCI‐AD and MCI‐LB groups were compared using chi‐squared test, Fisher exact test, Mann–Whitney *U*‐test, and *t*‐tests where appropriate. As there were more males in the MCI‐LB group than the MCI‐AD group, and longer time of follow‐up, comparison of cumulative frequency of symptoms was tested using logistic regression with sex and years of follow‐up as covariates. As 43 symptoms were tested, correction for multiple comparisons was carried out using the method of Benjamini and Yekutieli [22]. In the cross‐sectional replication comparison, area under the receiver operating characteristic curve (AUROC) was plotted to determine the ability of the 10‐point symptom score to discriminate between MCI‐LB and MCI‐AD [11].

## RESULTS

### Longitudinal cumulative prevalence

The group demographics and cumulative prevalence of each symptom are displayed in Table 1. The MCI‐LB group had longer follow‐up and a greater proportion were males when compared with MCI‐AD.

**TABLE 1:** Longitudinal cumulative frequency of symptoms in controls, MCI‐AD, and MCI‐LB.

Characteristic	Control	MCI‐AD	MCI‐LB	*p* (MCI‐AD vs. MCI‐LB)	*p*_adj_
*n*	34	51	70	n/a	
Age (SD)	74.2 (7.5)	76.4 (7.4)	75.2 (6.8)	0.38	
Sex, % male	71%	37%	77%	**<0.001**	
ACE total (SD)	92.7 (4.3)	81.4 (9.7)	80.6 (9.3)	0.65	
Duration of follow‐up, years (SD)	1.2 (0.7)	1.5 (1.5)	2.0 (1.4)	**0.02**	
Cognitive symptoms					
Memory	24%	100%	99%	1	1
Problem‐solving	3%	31%	67%	0.002	**0.02**
Planning	0%	33%	69%	0.002	**0.02**
Fluctuations	12%	25%	69%	0.001	**0.02**
Disorganized speech	3%	16%	46%	0.01	0.08
Confusion	3%	12%	44%	<0.001	**0.01**
Symptoms associated with Parkinson disease					
Rigidity	12%	18%	43%	0.23	1
Shuffling	6%	16%	69%	<0.001	**0.002**
Tremor	15%	35%	67%	0.002	**0.02**
Slowness	9%	55%	77%	0.14	0.73
Change in writing	18%	45%	83%	0.001	**0.01**
Slack facial expression	0%	10%	34%	0.12	0.62
Drooling	12%	16%	51%	0.003	**0.03**
Loss of smell	21%	31%	60%	0.02	0.14
Balance	24%	43%	73%	0.007	0.06
Frequent falls	6%	20%	36%	0.07	0.41
Posture	24%	49%	86%	0.001	**0.02**
Weak voice	6%	25%	53%	0.07	0.41
Neuropsychiatric symptoms					
Seeing things	0%	18%	57%	<0.001	**0.01**
Hearing things	0%	4%	27%	0.002	**0.02**
Depression	3%	27%	47%	0.13	0.66
Apathy	3%	37%	70%	0.01	0.11
Delusions	0%	2%	13%	0.02	0.16
Hallucinations other senses	6%	12%	27%	0.01	0.08
Anxiety	6%	41%	54%	0.70	1
Sleep symptoms					
Vivid dreams	3%	20%	57%	0.003	**0.03**
Nightmares	0%	6%	50%	<0.001	**0.01**
Involuntary movements in sleep	3%	10%	66%	<0.001	**0.01**
Acting out dreams	0%	6%	57%	<0.001	**0.01**
Crying out	6%	20%	64%	0.002	**0.02**
Excessive sleepiness	6%	29%	67%	0.001	**0.02**
Blackouts	0%	10%	7%	0.77	1
Insomnia	9%	22%	33%	0.03	0.20
Restless legs	12%	20%	26%	0.54	1
Autonomic symptoms					
Dizziness	29%	29%	61%	0.006	0.06
Sensitivity to heat/cold	44%	47%	84%	<0.001	**0.01**
Sexual dysfunction	24%	34%	72%	0.24	1
Urinary incontinence	12%	33%	53%	0.05	0.32
Constipation	18%	33%	54%	0.03	0.22
Visual symptoms					
Dry/painful eyes	32%	31%	50%	0.08	0.46
Double vision	6%	12%	30%	0.05	0.32
Difficulty reading	3%	12%	36%	0.01	0.06
Misjudging objects	9%	10%	53%	<0.001	**0.01**

*Note*: Logistic regression with sex and years of follow‐up as covariates. Bold indicates statistical significance. Sexual dysfunction, *n* = 124; for all other variables *n* = 155. Abbreviations: ACE, Addenbrooke Cognitive Examination; MCI, mild cognitive impairment; MCI‐AD, MCI due to Alzheimer disease; MCI‐LB, mild cognitive impairment with Lewy bodies; n/a, not applicable; *p*_adj_: adjusted *p*‐value following false discovery rate correction.

The following symptoms were significantly more common in MCI‐LB than MCI‐AD after including sex and years of follow‐up as covariates in logistic regression:

Cognitive symptoms: problem‐solving, planning, fluctuations, confusion.
Parkinsonian symptoms: shuffling, tremor, change in writing, drooling, change in posture.
Neuropsychiatric: seeing and hearing things that are not present.
Sleep symptoms: vivid dreams, nightmares, involuntary movements in sleep, acting out dreams, crying out in sleep, excessive sleepiness.
Autonomic symptoms: sensitivity to heat and cold.
Visual symptoms: misjudging objects.

Of the 43 symptoms, MCI‐LB participants experienced a mean (SD) of 24.2 (7.6) symptoms, compared with 11.3 (7.4) for MCI‐AD and 4.2 (3.7) for controls (*p* < 0.001 for all comparisons with sex and years of follow‐up as covariates).

Although many symptoms were more common in MCI‐LB than MCI‐AD, when present, the time of onset of symptoms was similar in the two groups (Table S2).

**FIGURE 1:**
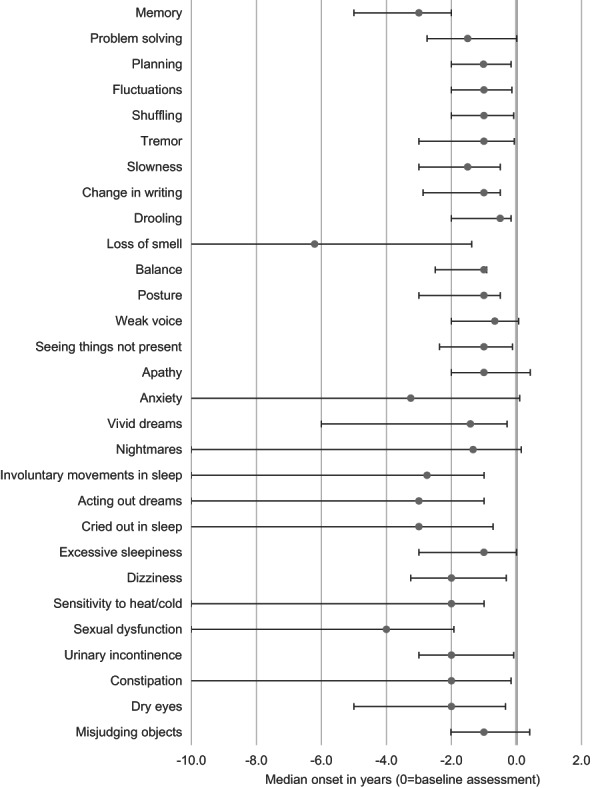
Temporal onset of symptoms in mild cognitive impairment with Lewy bodies. Grey points: median; bars: interquartile range. Symptoms present in ≥50% of participants with mild cognitive impairment with Lewy bodies are shown.

A scatterplot of symptom onset and cumulative frequency in MCI‐LB is displayed in Supplementary Figure S1. The onset of common symptoms in MCI‐LB (cumulative prevalence > 50%) is displayed in Figure 1. Most symptoms had a median time of onset within 2 years prior to baseline assessment, and later than the first cognitive symptoms. Sleep symptoms (involuntary movements in sleep, acting out dreams, and crying out in sleep), sexual dysfunction, loss of smell, memory problems, and anxiety had a median time of onset of >2 years before baseline assessment.

### Cross‐sectional replication of 10‐symptom discrimination

The demographics of the cohort and the prevalence of each symptom in the diagnostic groups are displayed in Table S3.

Ten symptoms were identified in our previous publication [11] as being relatively sensitive (>50%) and specific (>80%) to DLB in comparison to AD (these are listed in Table 2), and these symptoms showed good discriminant ability between MCI‐LB and MCI‐AD in our previous cohort. This was analysed in the participants in this cohort who were not part of the original publication. The mean number of symptoms in MCI‐LB was greater than MCI‐AD (median = 4, interquartile range [IQR] = 2.5–5 vs. 0, IQR = 0–1; *p* < 0.001). The AUROC for this 10‐point scale was 0.91 (95% confidence interval = 0.84–0.98; Figure S2). Good discrimination between MCI‐LB and MCI‐AD was demonstrated for threshold scores of >1 (sensitivity = 83%, specificity = 83%), >2 (sensitivity = 76%, specificity = 90%), and >3 (sensitivity = 59%, specificity = 97%).

**TABLE 2:** Ten‐symptom list tested for discriminant ability.

Fluctuating concentration/attention
Episodes of confusion
Slack facial expression
Drooling
Weak voice
Seeing things not present
Involuntary movements
Acting out dreams
Crying out during sleep
Misjudging objects

## DISCUSSION

This study presents a large cohort of MCI‐LB with a mean of 2 years of longitudinal follow‐up, giving a clear indication of the symptom burden experienced by people with MCI‐LB, their time of onset, and the ability of these symptoms to discriminate between MCI‐LB and MCI‐AD. In the following discussion, we will make a clear distinction between overall symptom burden, which is important in the clinical management of symptoms and quality of life in people with MCI‐LB, and discriminant ability, which is useful in clinical diagnosis.

### Clinical relevance: Symptom burden

Participants with MCI‐LB experienced a very high symptom burden, with an average of 24 of 43 symptoms in each participant. Many symptoms were common to both MCI‐LB and MCI‐AD, but this does not detract from their potential clinical significance. MCI‐LB presents with a range of cognitive symptoms, but almost all people with MCI‐LB reported memory problems. Motor parkinsonism, RBD, and visual experiences were, by definition, common in the MCI‐LB group. However, the range of symptoms in MCI‐LB extended far beyond these core clinical features.

The following symptoms, which are not core clinical features or features of cognitive dysfunction, were experienced by most people with MCI‐LB: drooling, loss of smell, balance problems, apathy, anxiety, vivid dreams/nightmares, excessive sleepiness, dizziness, sensitivity to heat/cold, sexual dysfunction, urinary incontinence, constipation, dry eyes, and misjudging objects. Some of these are potentially treatable, particularly autonomic symptoms [23].

Memory services should ensure that cognitive, motor, neuropsychiatric, sleep, autonomic, and visual symptoms are enquired about during assessment and take opportunities to target potentially treatable symptoms such as constipation, incontinence, drooling, and sexual dysfunction.

There were no significant differences between MCI‐LB and MCI‐AD in timing of symptom onset. It is important to note that many noncognitive symptoms in MCI‐LB have a time of onset soon after the development of first cognitive symptoms. Therefore, the initial memory assessment presents an excellent opportunity to screen for these symptoms.

### Clinical relevance: Discriminant ability

Core clinical features are less common in MCI‐LB than DLB, and diagnostic biomarkers also have lower sensitivity at the MCI stage [11, 12, 24]. Consideration of supportive clinical features has the potential to aid clinical diagnosis.

As expected, symptoms associated with the core clinical features of MCI‐LB were more common in MCI‐LB than MCI‐AD. The following features were also significantly more common in MCI‐LB: difficulties with planning and problem‐solving, drooling, hearing things not present, vivid dreams, nightmares, excessive sleepiness, sensitivity to heat/cold, and misjudging objects. These symptoms could be considered during clinical assessment in addition to core clinical features, to help improve the detection of MCI‐LB.

Our findings are in keeping with previous reports, which have identified high rates of neuropsychiatric, motor, sleep, and autonomic symptoms in MCI‐LB or prodromal DLB [6, 7, 8, 9, 10]. The control group in our study had higher rates of some symptoms in comparison to other cohorts, such as constipation [6] and drooling/hypersalivation [9]. We recruited MCI participants with possible symptoms related to prodromal DLB; therefore, the rates of the symptoms reported in MCI‐AD here may be higher than in the general clinical population. Some symptoms have high variation in prevalence globally, for example, constipation [25]. This highlights the importance of understanding the rates of symptoms within local populations when applying clinical scales.

Importantly, constipation and loss of smell were relatively common in MCI‐AD (>30%) and should not be considered specific to MCI‐LB. A significant difference between MCI‐LB and MCI‐AD in constipation has been reported in another cohort. However, constipation was still relatively common in MCI‐AD (62% vs. 21%) [6]. This study also found obstipation (severe constipation) to be more common in MCI‐LB than MCI‐AD (43% vs. 15%). Differences have been noted between MCI‐LB and MCI‐AD in direct testing of olfactory function, which may be more effective than patient/carer report of hyposmia [26, 27].

### Research relevance: Symptom burden

The scale of the symptom burden experienced in MCI‐LB is highly significant and is consistent with data published by other groups on the prevalence of autonomic, neuropsychiatric, and other symptoms in MCI‐LB [6, 7, 8, 9, 10]. These symptoms are also common in DLB. Few studies have investigated the biological basis of important symptoms such as apathy and anxiety in MCI‐LB or DLB, or attempted to test symptomatic treatments. Given the prevalence of these symptoms and the distress associated with them, research in this area is urgently needed.

### Research relevance: Discriminant ability

We replicated a previous finding that a brief list of 10 symptoms can accurately differentiate between MCI‐AD and MCI‐LB. We do not currently recommend the use of such a questionnaire as a diagnostic tool in the clinic, where clinical expertise and application of current criteria are a more appropriate approach. However, in research settings, it may be valuable to enrich cohorts with MCI‐LB cases using a simple questionnaire that has the potential to be used remotely, by nonexpert diagnosticians and in a large number of potential participants. The appropriate threshold would depend on the sensitivity and specificity required for the particular research application.

### Strengths and limitations

This article presents a clinically well‐characterized MCI cohort with biomarker support for MCI‐LB diagnosis from the LewyPro [11] and SUPErB [14] studies. This paper reports baseline cross‐sectional symptom frequency data from the SUPErB study and combined longitudinal cumulative frequency from both the SUPErB and LewyPro studies. The LewyPro study cross‐sectional data have previously been reported [11], and in the current paper we have replicated the discriminant ability of a 10‐point symptom scale developed using data from that cohort. The cross‐sectional data reported now only include participants who were not part of the LewyPro study; therefore, there is no overlap in the cross‐sectional data presented here and that of our previous paper. The cognitive profile of the cohorts has been published previously [13, 14, 28].

The cohort of MCI‐LB is large compared to most published cohorts and presents a broader range of symptoms than other publications that have described the clinical presentation of MCI‐LB. The MCI‐AD group was smaller than the MCI‐LB group. A larger MCI‐AD group may have increased the number of symptoms, demonstrating a statistically significant difference between the two groups. The longitudinal follow‐up allows us to report the total burden of symptoms over the course of MCI. However, longer follow‐up may have identified more participants with less commonly reported clinical symptoms, such as hallucinations or delusions, prior to dementia conversion. In addition, participants and informants may not accurately recall the time of onset of symptoms. Longitudinal studies that recruit participants in the pre‐MCI stages of disease would provide a more accurate description of symptom onset. Unfortunately, at present there is no effective method to identify and recruit presymptomatic cases of DLB to clinical studies. This contrasts with AD, where the presence of AD pathology can be determined by CSF analysis or positron emission tomography (PET) amyloid/tau imaging prior to the onset of symptoms.

We excluded participants with a history of clinical stroke; therefore, we cannot comment on the discriminant ability of these symptoms when differentiating MCI‐LB from vascular MCI. Participants did not have CSF or PET measures of amyloid or tau; therefore, we are unable to comment on the effects of AD pathology on clinical symptoms in MCI‐LB. We have previously investigated the association of amyloid deposition with clinical profile in the dementia stage of DLB. Although amyloid deposition was associated with more rapid cognitive decline, it was not associated with differences in clinical symptoms [29, 30].

Most participants had an informant present, which increases the reliability of symptom report, but we cannot comment on the reliability of the questionnaire without an informant present.

The presence of RBD was determined clinically, as defined in the MCI‐LB criteria [3]. Participants did not have polysomnography to confirm the presence of REM sleep without atonia as part of the study. Although there is a risk that other sleep disorders may mimic RBD, it should be noted that, to qualify for a diagnosis of probable MCI‐LB, at least one other core clinical feature or a positive biomarker must be present. As we have previously published, the majority of MCI‐LB cases had a positive biomarker (i.e., abnormal striatal dopaminergic imaging, abnormal cardiac MIBG scintigraphy, or both) [12, 24].

Control of the false discovery rate (FDR) was carried out using the method of Benjamini and Yekutieli [22]. This method is more conservative than other methods of FDR correction (e.g., Benjamini and Hochberg [31]). Importantly, this method remains valid when there is dependency in *p*‐values (as would be expected in this analysis). The MCI‐LB and MCI‐AD groups differed in proportion of males and duration of follow‐up, but these variables were included as covariates in the statistical analysis. Despite the substantial sample size, some symptoms appeared approach statistical significance, and it is likely they would be significant in a larger sample. Nevertheless, the sample size is likely sufficient to detect differences that will be clinically relevant.

There is a possibility that the presence of core features such as visual hallucinations may have made interviewers more likely to endorse other symptoms thought to be related to DLB. However, interviewers were instructed to accept the participant's answer without interpretation, and the relatively low prevalence of symptoms previously thought to be strongly associated with Lewy body disease (e.g., constipation) suggests that this was successful. Diagnostic raters were not blind to the symptom questionnaire; however, more detailed, symptom‐specific scales were used to identify the presence or absence of core clinical features.

## CONCLUSIONS

MCI‐LB is associated with a high prevalence of a range of cognitive, motor, neuropsychiatric, sleep, autonomic, and visual symptoms. A range of symptoms in MCI‐LB have their onset soon after the first cognitive symptoms. Therefore, the first presentation to a memory assessment service is a good opportunity to identify these symptoms, explain their cause, and where appropriate, offer treatment or onward referral.

Clinicians should be aware of the prevalence of these symptoms, the potential for treatment, and their discriminant ability to identify MCI‐LB. Future research should validate the 10‐point symptom scale in unselected clinic‐based populations and identify effective symptomatic treatments for common symptoms such as anxiety and apathy in MCI‐LB.

## AUTHOR CONTRIBUTIONS

**Paul C. Donaghy:** Conceptualization; formal analysis; investigation; methodology; supervision; writing–original draft. **Calum Hamilton:** Conceptualization; data curation; investigation; methodology; writing–review and editing. **Rory Durcan:** Data curation; investigation; writing–review and editing. **Sarah Lawley:** Data curation; investigation; writing–review and editing. **Sally Barker:** Data curation; investigation; writing–review and editing. **Joanna Ciafone:** Data curation; investigation; writing–review and editing. **Nicola Barnett:** Data curation; investigation; writing–review and editing. **Kirsty Olsen:** Data curation; investigation; writing–review and editing. **Michael Firbank:** Conceptualization; data curation; writing–review and editing. **Gemma Roberts:** Data curation; investigation; writing–review and editing. **Jim Lloyd:** Conceptualization; writing–review and editing. **Louise M. Allan:** Conceptualization; writing–review and editing. **Ranjan Saha:** Data curation; writing–review and editing. **Ian G. McKeith:** Conceptualization; writing–review and editing. **John T. O'Brien:** Conceptualization; writing–review and editing. **John‐Paul Taylor:** Conceptualization; investigation; writing–review and editing. **Alan J. Thomas:** Conceptualization; funding acquisition; investigation; methodology; project administration; supervision; writing–review and editing.

## Funding information

This work was supported by Alzheimer's Research UK (A.J.T., grant number ARUK‐PG3026‐13) and the NIHR Newcastle Biomedical Research Centre. GE Healthcare provided funding for FP‐CIT imaging for this investigator‐led study. P.C.D. is supported by the Medical Research Council (grant number MR/W000229/1). L.M.A. was supported by the National Institute for Health Research Applied Research Collaboration South West Peninsula. J.T.O. is supported by the NIHR Cambridge Biomedical Research Centre. The views expressed in this publication are those of the authors and not necessarily those of the National Institute for Health Research or the Department of Health and Social Care.

## CONFLICT OF INTEREST STATEMENT

G.R. has delivered educational presentations at workshops organized by GE Healthcare, for which her employer (Newcastle Upon Tyne Hospitals NHS Foundation Trust) receives payment. J.T.O. has provided consultancy advice and delivered educational workshops for GE Healthcare. J.‐P.T. has delivered educational workshops for GE Healthcare. None of the other authors has any conflict of interest to disclose.

## ETHICAL APPROVAL

The authors assert that all procedures contributing to this work comply with the ethical standards of the relevant national and institutional committees on human experimentation and with the Helsinki Declaration of 1975, as revised in 2008.

## Supporting information


**TABLES S1** **–S3**
FIGURES S1–S2

## Data Availability

The data that support the findings of this study are available from the corresponding author upon reasonable request.
