# Supplementary material for: Clinical symptoms in mild cognitive impairment with Lewy bodies: Frequency, time of onset, and discriminant ability
Source: Eur J Neurol. 2023 Mar 21;30(6):1585–93. doi: 10.1111/ene.15783 (PMC10946617; doi:10.1111/ene.15783)
Supplement: Supplementary file 1 — TABLES S1 –S3 FIGURES S1–S2 [file ENE-30-1585-s001.pdf]

Supplementary Table 1. The Symptom Questionnaire

**Cognitive Symptoms**<br>Memory<br>Problem Solving<br>Planning<br>Fluctuating changes in concentration and attention<br>Disorganised speech and conversation<br>Unexplained episodes of confusion<br><br>**Parkinson's Symptoms**<br>Rigidity or stiffness in muscles<br>Shuffling walk<br>Tremor<br>Slowness of movement<br>Change in Handwriting<br>Slack facial expression<br>Drooling<br>Loss/reduction of sense of smell<br>Balance problems<br>Frequent falls<br>Change in posture<br>Weak voice<br><br>**Behaviour/mood changes**<br>Seeing things that are not present<br>Hearing things that are not present<br>Depression<br>Apathy (loss of interest and drive)<br>Delusions (false beliefs)<br>Hallucinations in other senses (e.g. touch or smell)<br>Anxiety	**Sleep Symptoms**<br>Troubled by vivid dreams<br>Troubled by nightmares<br>Had involuntary movements of arms and legs<br>Acting out dreams, sometimes violently<br>Cried out during sleep<br>Excessive daytime sleepiness<br>Transient loss of consciousness/unexplained blackouts<br>Insomnia<br>Restless legs syndrome<br><br>**Autonomic Dysfunction**<br>Dizziness, light-headedness or fainting<br>Sensitivity to heat or cold<br>Sexual Dysfunction<br>Urinary incontinence<br>Constipation<br><br>**Visual Symptoms**<br>Painful/dry eyes<br>Double vision<br>Difficulty reading (because words and letters move around the page)<br>Misjudging objects (have difficulty moving around because you misjudge where objects are)

Supplementary Table 2. Time of onset of symptoms in MCI-AD and MCI-LB

	Onset (median (IQR)) relative to initial assessment		
	**MCI-AD**	**MCI-LB**	**p**
Cognitive symptoms			
Memory	-2.0 (-3.5, -2.0)	-3.0 (-5.0, -2.0)	0.16
Problem Solving	-1.0 (-2.8, -0.7)	-1.5 (-2.8, 0.0)	0.67
Planning	-1.5 (-4.2, 0.1)	-1.0 (-2.0, -0.2)	0.43
Fluctuations	-1.5 (-4.0, 0.3)	-1.0 (-2.0, -0.1)	0.48
Disorganised Speech	-1.5 (-3.7, -0.3)	-0.5 (-2.0, 0.5)	0.16
Confusion	0.8 (-1.3, 1.6)	-0.8 (-1.7, 0.8)	0.36
Symptoms associated with Parkinson's disease			
Rigidity	0.0 (-2.8, 0.2)	-0.8 (-2.8, 0.3)	0.61
Shuffling	-1.5 (-3.1, 0.4)	-1.0 (-2.0, -0.1)	0.49
Tremor	-1.5 (-6.5, -0.5)	-1.0 (-3.0, -0.1)	0.18
Slowness	-2.0 (-4.8, -0.4)	-1.5 (-3.0, -0.5)	0.78
Change in Writing	-2.9 (-6.0, -1.0)	-1.0 (-2.9, -0.5)	<.01
Slack facial exp.	0.4 (-2.0, 0.9)	-0.2 (-1.4, 0.5)	0.93
Drooling	-0.1 (-0.9, 0.6)	-0.5 (-2.0, -0.2)	0.07
Loss of smell	-2.0 (-21.3, -1.0)	-6.2 (-17.1, -1.4)	0.53
Balance problems	-1.5 (-4.5, -0.2)	-1.0 (-2.5, -0.9)	0.59
Frequent Falls	0.4 (-1.3, 0.6)	-1.5 (-2.5, -0.3)	0.13
Change in posture	-1.0 (-5.0, 0.0)	-1.0 (-3.0, -0.5)	0.61
Weak Voice	-1.8 (-3.0, -0.0)	-0.7 (-2.0, 0.1)	0.33
Neuropsychiatric symptoms			
Seeing things	-0.6 (-1.4, 0.6)	-1.0 (-2.4, -0.1)	0.44
Hearing things	n/a	-0.5 (-2.0, 0.7)	0.84
Depression	-2.5 (-42.5, 0.6)	-2.0 (-5.0, 0.5)	0.47
Apathy	-1.0 (-2.0, 0.1)	-1.0 (-2.0, 0.4)	0.93
Delusions	n/a	0.0 (-2.9, 0.7)	0.40
Hallucinations other senses	-0.4 (-1.2, 0.3)	-0.1 (-1.0, 0.9)	0.72
Anxiety	-2.0 (-20.5, 0.1)	-3.3 (-40.0, 0.1)	0.49
Sleep symptoms			
Vivid Dreams	-1.0 (-4.8, 0.0)	-1.4 (-6.0, -0.3)	0.68
Nightmares	n/a	-1.3 (-10.0, 0.1)	0.54
Involuntary movements in sleep	-3.9 (-6.5, 0.6)	-2.8 (-10.0, -1.0)	0.38
Acting out Dreams	n/a	-3.0 (-10.0, -1.0)	0.12
Cried Out	-1.0 (-4.4, 0.3)	-3.0 (-15.0, -0.7)	0.06
Excessive sleepiness	-1.5 (-3.9, -0.1)	-1.0 (-3.0, 0.0)	0.60
Blackouts	-3.0 (-9.3, 0.3)	n/a	0.22
Insomnia	-1.3 (-23.8, -0.1)	-2.3 (-7.0, -0.1)	0.91
Restless legs	-1.5 (-46.3, 1.2)	-5.5 (-15.0, -1.5)	0.86
Autonomic symptoms			
Dizziness	-2.0 (-7.0, -0.9)	-2.0 (-3.3, -0.3)	0.64

Sensitivity to heat/cold	-3.0 (-20.0, -0.5)	-2.0 (-10.0, -1.0)	1.00
Sexual Dysfunction	-2.4 (-9.3, -1.1)	-4.0 (-10.0, -1.9)	0.68
Urinary incontinence	-2.0 (-7.5, 0.0)	-2.0 (-3.0, -0.1)	0.59
Constipation	-6.5 (-60.5, -1.6)	-2.0 (-12.5, -0.2)	0.09
Visual symptoms			
Dry eyes	-3.0 (-4.3, -1.6)	-2.0 (-5.0, -0.3)	0.66
Double vision	-0.3 (-10.6, 2.3)	-1.0 (-3.0, -0.3)	0.37
Difficulty Reading	-2.0 (-25.5, 0.2)	-0.7 (-1.9, 1.6)	0.17
Misjudging Objects	-1.0 (-7.5, 3.1)	-1.0 (-2.0, 0.4)	0.86
Time of onset relative to baseline assessment in years. Time of onset not stated if present in less than 10% of participants. P=uncorrected p value. MCI mild cognitive impairment; AD Alzheimer's disease; LB Lewy bodies.			

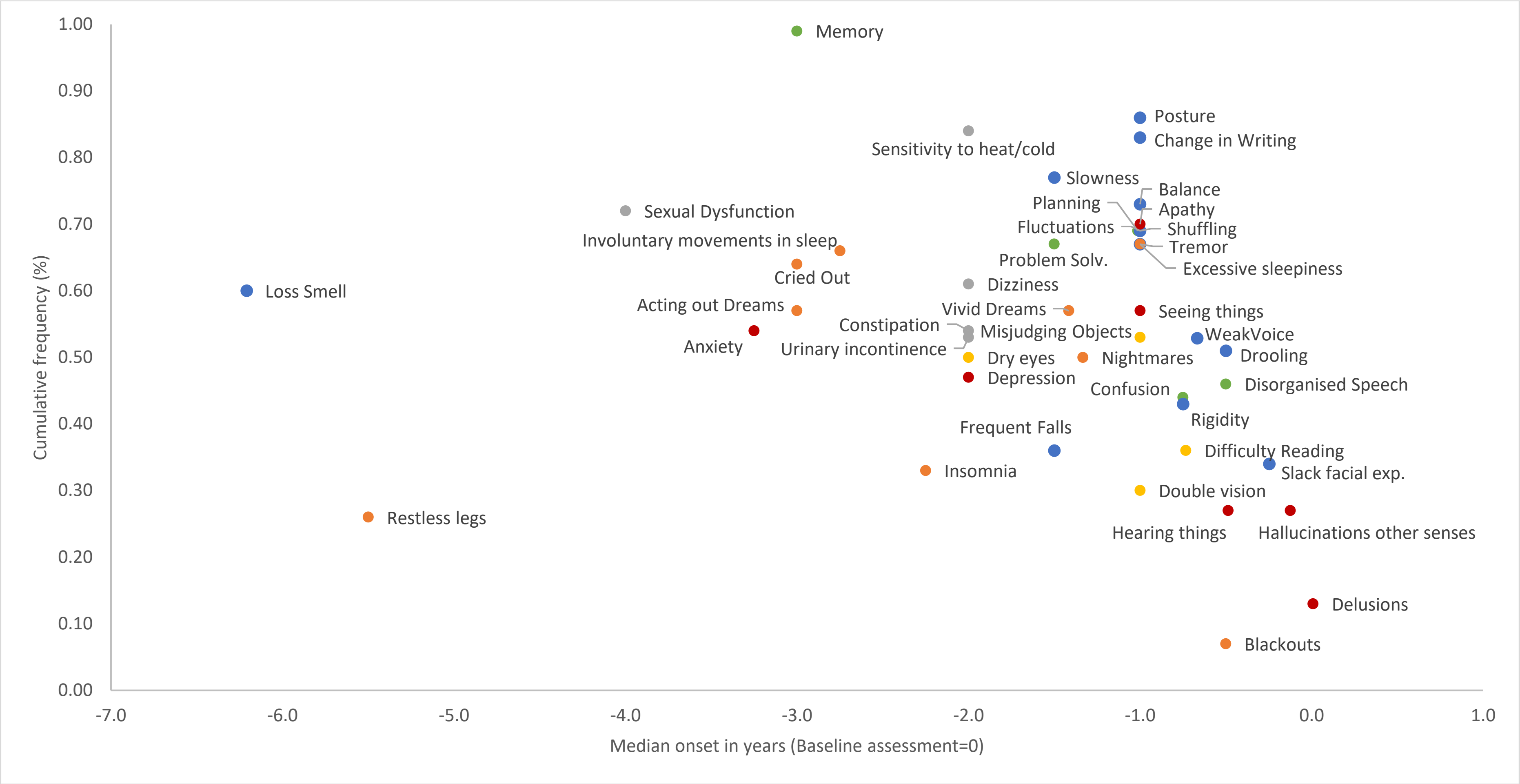

Supplementary Figure 1. Scatterplot of median symptom onset and cumulative prevalence in MCI-LB. Green – cognitive symptoms; blue – symptoms associated with Parkinson's disease; red – neuropsychiatric symptoms; orange – sleep symptoms; grey – autonomic symptoms; yellow – visual symptoms.

Supplementary Table 3: Baseline demographics and symptoms prevalence in the SUPErB cohort

	Control	MCI-AD	MCI-LB	p (MCI-AD v MCI-LB)
N	33	29	29	
Age	74.3 (7.5)	74.9 (7.1)	74.2 (6.0)	0.92
Sex, n (% male)	24 (73)	11 (38)	27 (83)	**<0.001**
ACE-R, mean (SD)	92.7 (4.3)	82.2 (8.2)	81.8 (10.0)	0.98
Informant present, n (%)	-	24 (83)	28 (97)	0.19
Live with informant, n (%)	-	16 (67)	26 (93)	**0.03**
AChI, n (%)	0 (0)	6 (21)	19 (66)	**0.001**
Levodopa, n (%)	0 (0)	0 (0)	2 (7)	0.49
Cognitive Symptoms				
Memory, n (%)	7 (21)	29 (100)	29 (100)	-
Problem Solving, n (%)	1 (3)	7 (24)	13 (45)	0.10
Planning, n (%)	0 (0)	4 (14)	12 (41)	**0. 02**
Fluctuations, n (%)	4 (12)	3 (10)	16 (57)	**<0.001**
Disorganized Speech, n (%)	0 (0)	1 (4)	9 (31)	**0.01**
Episodes of Confusion, n (%)	1 (3)	1 (3)	4 (15)	0.19
Symptoms associated with Parkinson's disease				
Rigidity or stiffness, n (%)	3 (9)	2 (7)	6 (21)	0.25
Shuffling walk, n (%)	1 (3)	2 (7)	13 (45)	**0.001**
Tremor, n (%)	5 (15)	7 (24)	13 (45)	0.10
Slowness of movement, n (%)	3 (9)	9 (31)	17 (59)	**0.04**
Change in writing, n (%)	5 (15)	12 (41)	19 (66)	0.07
Slack facial expression, n (%)	0 (0)	1 (4)	5 (17)	0.19
Drooling, n (%)	2 (6)	4 (14)	11 (38)	**0.04**
Loss of smell, n (%)	5 (15)	8 (28)	15 (52)	0.06
Balance problems, n (%)	5 (15)	9 (31)	16 (59)	**0.03**
Frequent falls, n (%)	0 (0)	2 (7)	4 (14)	0.67
Change in posture, n (%)	6 (18)	9 (31)	23 (79)	**<0.001**
Weak voice, n (%)	2 (6)	6 (21)	12 (41)	0.09
Neuropsychiatric symptoms				
Seeing things, n (%)	0 (0)	2 (7)	7 (24)	0.14
Hearing things, n (%)	0 (0)	0 (0)	4 (14)	0.11
Depression, n (%)	0 (0)	3 (10)	9 (31)	0.05
Apathy, n (%)	0 (0)	6 (21)	10 (35)	0.24
Delusions, n (%)	0 (0)	0 (0)	1 (4)	1
Other Hallucinations, n (%)	2 (6)	1 (3)	5 (17)	0.19
Anxiety, n (%)	2 (6)	8 (28)	12 (43)	0.23
Sleep symptoms				
Vivid dreams, n (%)	0 (0)	2 (7)	15 (52)	**<.001**
Nightmares, n (%)	0 (0)	1 (3)	11 (38)	**0.001**
Involuntary movements, n (%)	1 (3)	1 (3)	20 (69)	**<0.001**
Acting out, n (%)	0 (0)	1 (3)	16 (55)	**<0.001**
Crying out, n (%)	1 (3)	2 (7)	16 (55)	**<0.001**
Excessive sleepiness, n (%)	1 (3)	5 (18)	12 (41)	0.05
Blackouts, n (%)	0 (0)	2 (7)	0 (0)	0.49
Insomnia, n (%)	1 (3)	7 (24)	3 (10)	0.16

Restless legs, n (%)	3 (9)	1 (3)	5 (18)	0.10
Autonomic symptoms				
Dizziness, n (%)	4 (12)	5 (17)	14 (48)	**0.01**
Sensitivity to heat or cold, n (%)	11 (33)	7 (25)	21 (72)	**<0.001**
Sexual dysfunction, n (%)	6 (23)	4 (22)	14 (56)	**0.03**
Urinary incontinence, n (%)	3 (9)	7 (25)	14 (48)	0.07
Constipation, n (%)	5 (15)	6 (21)	8 (28)	0.54
Visual Symptoms				
Dry/painful eyes, n (%)	10 (30)	7 (24)	9 (32)	0.50
Double vision, n (%)	1 (3)	2 (7)	4 (14)	0.42
Difficulty reading, n (%)	1 (3)	4 (14)	8 (29)	0.19
Misjudging objects, n (%)	3 (9)	3 (10)	10 (37)	**0.02**
Sexual dysfunction n=69, for all other variables n=89-91 (occasionally participants felt unable to answer). Bold: significant p<.05 (uncorrected). MCI mild cognitive impairment; AD Alzheimer's disease; LB Lewy bodies.				

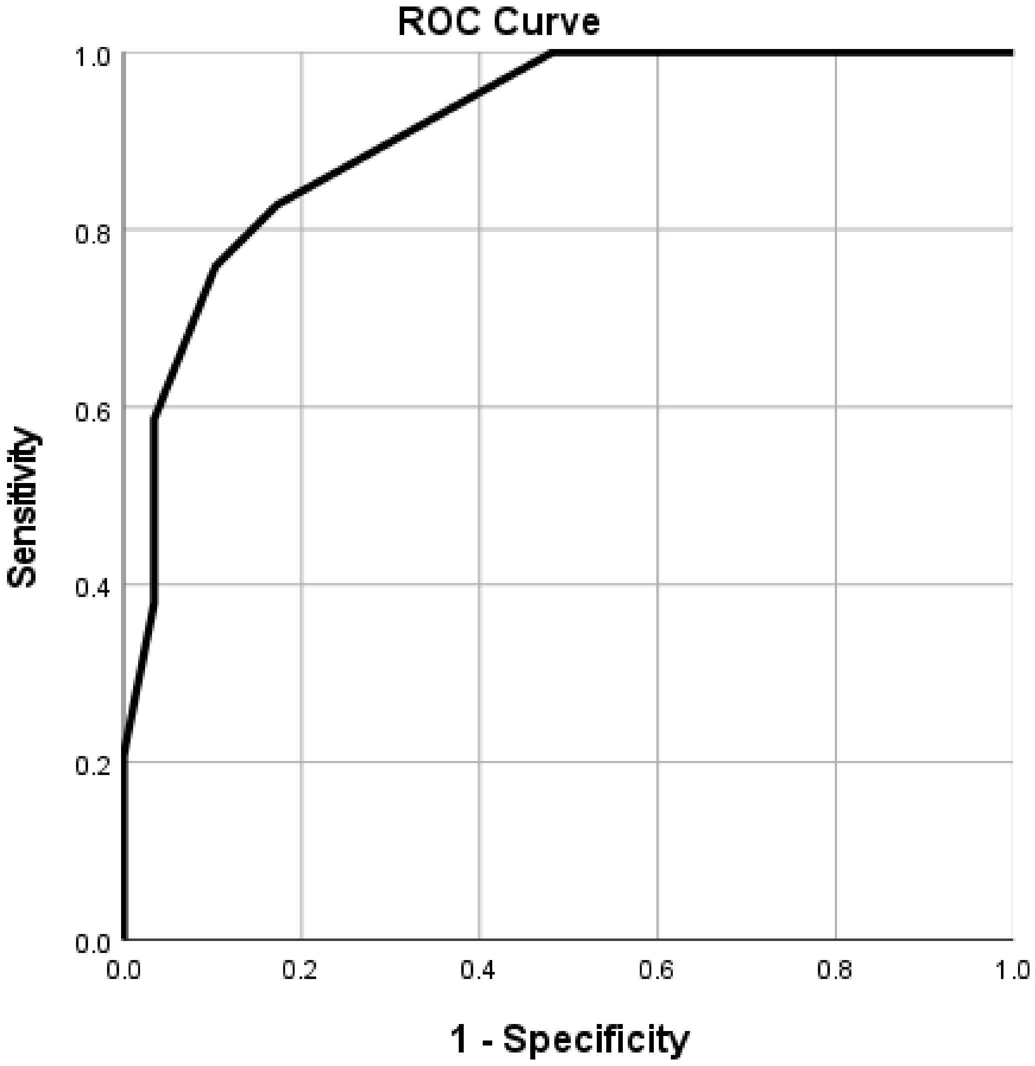

Supplementary Figure 2. Receiver Operating Characteristic for the 10-point symptom scale to differentiate MCI-LB from MCI-AD. Area under ROC 0.91 (95% CI 0.84-0.98).
